# Biguanides enhance antifungal activity against *Candida glabrata*

**DOI:** 10.1080/21505594.2018.1475798

**Published:** 2018-08-01

**Authors:** Shuying Xu, Marianela Feliu, Allison K. Lord, Daniel P. Lukason, Paige E. Negoro, Nida S. Khan, Zeina Dagher, Michael B. Feldman, Jennifer L. Reedy, Samantha N. Steiger, Jenny M. Tam, Alexander A. Soukas, David B. Sykes, Michael K. Mansour

**Affiliations:** a Division of Infectious Disease, Massachusetts General Hospital, Boston, MA, USA; b Biomedical Engineering and Biotechnology, University of Massachusetts Medical School, Worcester, MA, USA; c Division of Pulmonary and Critical Care, Massachusetts General Hospital, Boston, MA, USA; d Department of Internal Medicine, Harvard Medical School, Boston, MA, USA; e Deparment of Pharmacy, Massachusetts General Hospital, Boston, MA, USA; f Diabetes Unit, Department of Endocrinology, Massachusetts General Hospital, Boston, MA, USA; g Center for Human Genetic Research, Massachusetts General Hospital, Boston, MA, USA; h Center for Regenerative Medicine, Massachusetts General Hospital, Boston, MA, USA

**Keywords:** *Candida*, *Candida glabrata*, biguanide, drug resistance, metformin, minimum inhibitory concentration (MIC)

## Abstract

*Candida spp*. are the fourth leading cause of nosocomial blood stream infections in North America. *Candida glabrata* is the second most frequently isolated species, and rapid development of antifungal resistance has made treatment a challenge. In this study, we investigate the therapeutic potential of metformin, a biguanide with well-established action for diabetes, as an antifungal agent against *C. glabrata*. Both wild type and antifungal-resistant isolates of *C. glabrata* were subjected to biguanide and biguanide-antifungal combination treatment. Metformin, as well as other members of the biguanide family, were found to have antifungal activity against *C. glabrata*, with MIC_50_ of 9.34 ± 0.16 mg/mL, 2.09 ± 0.04 mg/mL and 1.87 ± 0.05 mg/mL for metformin, phenformin and buformin, respectively. We demonstrate that biguanides enhance the activity of several antifungal drugs, including voriconazole, fluconazole, and amphotericin, but not micafungin. The biguanide-antifungal combinations allowed for additional antifungal effects, with fraction inhibition concentration indexes ranging from 0.5 to 1. Furthermore, metformin was able to lower antifungal MIC_50_ in voriconazole and fluconazole-resistant clinical isolates of *C. glabrata*. We also observed growth reduction of *C. glabrata* with rapamycin and an FIC of 0.84 ± 0.09 when combined with metformin, suggesting biguanide action in *C. glabrata* may be related to inhibition of the mTOR complex. We conclude that the biguanide class has direct antifungal therapeutic potential and enhances the activity of select antifungals in the treatment of resistant *C. glabrata* isolates. These data support the further investigation of biguanides in the combination treatment of serious fungal infections.

## Introduction

Invasive fungal infections due to non-*Candida albicans* species have been on the rise for the past two decades [1,2]. *Candida glabrata*, the most frequently isolated non-*C. albicans* species, accounts for around 21% of all *Candida*-related systemic bloodstream infections in North America [3]. The rise in number of *C. glabrata* infections is particularly concerning, due to the high antifungal resistance of this species. Unlike most other *Candida* bloodstream infections, *C. glabrata* is intrinsically less susceptible to antifungal agents [4]. This feature is especially true for the most widely used antifungal agent, fluconazole, with more than 10% of bloodstream infection isolates of *C. glabrata* showing high fluconazole resistance (MIC ≥ 64 μg/mL) [5]. *C. glabrata* also demonstrates rapid secondary antifungal resistance, which is suspected to be related to *C. glabrata* haploid genome [6].

A strategy to counter fluconazole-resistant infections is to use advanced and more potent synthetic derivative azole compounds. Voriconazole, for instance, is an azole whose antifungal spectrum is broader than fluconazole, with a potency 10 to 100 times greater against *Candida* species [7,8]. However, *C. glabrata* has rapidly developed resistance to voriconazole and other later generation azoles, presenting with higher MICs than those seen for most *C. albicans* isolates [6]. The current first-line treatment for candidemia due to *C. glabrata* are echinocandin agents, such as micafungin, which are potent inhibitors of beta-glucan synthase, a critical component of the fungal cell wall [1,9]. Echinocandins are efficacious against fluconazole-resistant *Candida* species, however, during prolonged therapy, *C. glabrata* can develop reduced susceptibility even to this class [10]. *C. glabrata* echinocandin resistance has increased from 4.9 to 12.3% between 2001 and 2010, and among the fluconazole-resistant isolates tested, 14.1% were fluconazole/echinocandin double-resistant [11]. The prevalence of drug resistance in *C. glabrata* is projected to increase with rise in susceptible patient populations, as use of immunosuppressive therapy and invasive surgical procedures expand, while older and more complex patient populations are becoming increasingly common [12–15]. Additionally, the use of broad spectrum antibiotics and suboptimal dosing practices are significant contributors to the rise of drug resistance [16]. These observations underscore the urgency to develop new antifungal strategies that may counter *C. glabrata* drug resistance.

Members of the biguanide drug class are defined by the common chemical feature of two guanidinium molecules joined by a single nitrogen, and include metformin, phenformin and buformin [17]. All three compounds exhibit anti-diabetic properties by reducing circulating glucose levels. Metformin, being the most well-established oral medication for management of type II diabetes, is prescribed as mono-therapy or in combination with other anti-hyperglycemic medicines [18–21]. The global usage of metformin is estimated to be 150 million individuals worldwide [22]. Recently, there has been interest surrounding the expanded therapeutic potential of metformin to include treatment and prevention of various types of cancer. Numerous clinical and preclinical studies suggest that metformin has anticancer properties, with a role in cancer stem cell suppression, epithelial-to-mesenchymal transition inhibition, and interference in cancer cell metabolism [23–25]. In addition, metformin is an effective adjuvant to several chemotherapy agents. A meta-analysis of clinical trials using metformin as an adjuvant demonstrated improved survival outcomes as compared with standard of care, especially in colorectal and prostate cancer patients [25].

Metformin is thought to exert its anticancer effects through inhibition of mammalian target of rapamycin complex 1 (mTORC1), which has crucial functions in metabolism, growth, and proliferation [26]. This metformin response pathway through mTORC1 is shown to be conserved across many organisms, from the nematode worms such as *Caenorhabditis elegans* to humans [27]. Despite these observations, there are no known observational studies suggesting that patients using metformin have lower fungal infections. We hypothesized that the same growth-inhibition pathway may also exist for fungal species and sought to test the potential of metformin as an antifungal agent.

Here, we describe the therapeutic potential of metformin against the fungal pathogen *C. glabrata* and demonstrate that multiple members of the biguanide family exhibit an antifungal effect. We show that in biguanide-antifungal combination therapies, biguanides enhance efficacy of antifungal agents across multiple classes. The antifungal properties of metformin extend to drug-resistant isolates of *C. glabrata*, and, importantly, can augment the therapeutic efficacy of antifungal drugs toward these difficult to treat infections.

## Results

### Metformin exhibits antifungal properties

**Figure 1.**
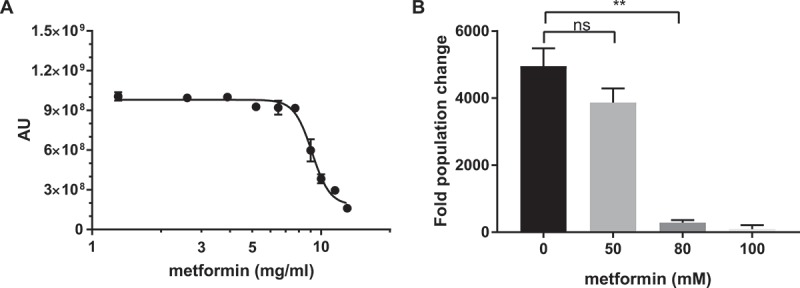
Metformin demonstrates antifungal activity against *C. glabrata*. (A) Prestoblue assay. Wild type *C. glabrata* were treated with a range of metformin concentrations, with Prestoblue fluorescence read at 18hrs. Arbitrary units (AU) for fluorescence plotted on the y-axis. Data points were fitted to a four-parameter logistic curve. (B) CFU assay. Wild type C. *glabrata* was treated with metformin for 18hrs, resuspended and plated onto YPD agar plates for 24-48hrs at 30°C. Data are plotted as fold population change compared to initial inoculum. ** denotes p ≤ 0.01, ns = not significant. Data represent 3 independent experiments.

To determine if metformin has antifungal properties towards *C. glabrata*, we inoculated *C. glabrata* into different concentrations of metformin, incubated for 20–24 h, and measured yeast viability using Prestoblue viability dye conversion as well as by CFU on YPD plates. We observed a decreased growth of *C. glabrata* in the presence of metformin with a MIC_50_ of 9.34 ± 0.16 mg/mL, using the Prestoblue viability dye conversion assay (Figure 1(A)). Similarly, there was a significant reduction in CFU at metformin concentrations of 10 mg/mL and higher (Figure 1(B)). Metformin is also effective against several other *Candida* species, including *C. albicans, C. krusei. C. tropicalis, and C. parapsilosis*, but not *C. auris*, although metformin sensitivity varies among the species (Table 1).

**Table 1.** MIC_50_ of various *Candida* species.

	MIC_50_(mg/mL)
C. glabrata	9.14 ± 0.18
C. albicans	20.41 ± 0.32
C. krusei	7.92 ± 0.53
C. tropicalis	14.57 ± 0.72
C. parapsilosis	15.39 ± 0.03
C. auris	≥ 25

**Figure 2.**
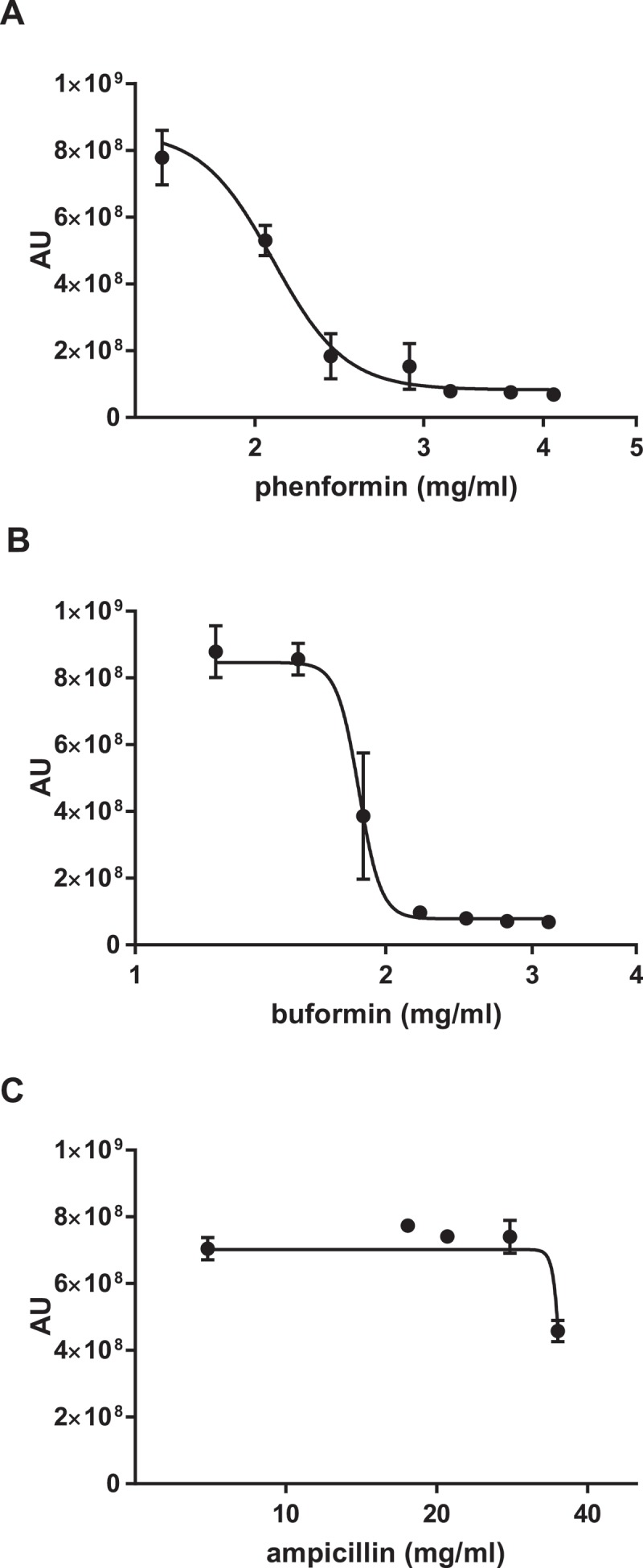
Antifungal activity is a shared feature of the biguanide family. Wild type *C. glabrata* were incubated with phenformin (A) or buformin (B) in the presence of Prestoblue for 18h at 30°C and arbitrary units for fluorescence determined (AU). (C) As a drug concentration control, *C. glabrata* was incubated with ampicillin. Data points were fitted to a four-parameter logistic curve and represent 3 independent experiments.

To determine whether this antifungal activity was unique to metformin or was a class effect of biguanides, we tested two additional commercially available biguanides. Phenformin and buformin, both more potent biguanides than metformin, also inhibited *C. glabrata* growth (Figure 2(A,B)). The MIC_50_ for phenformin was 2.09 ± 0.04 mg/mL, and for buformin 1.87 ± 0.05 mg/mL. To control for the high biguanide concentrations required in this study, we used the antibacterial agent ampicillin as a chemical concentration control for non-specific drug effect. At concentrations that the biguanides, including metformin, were effective in reducing *C. glabrata* growth, ampicillin did not exhibit any effect (Figure 2(C)).

**Figure 3.**
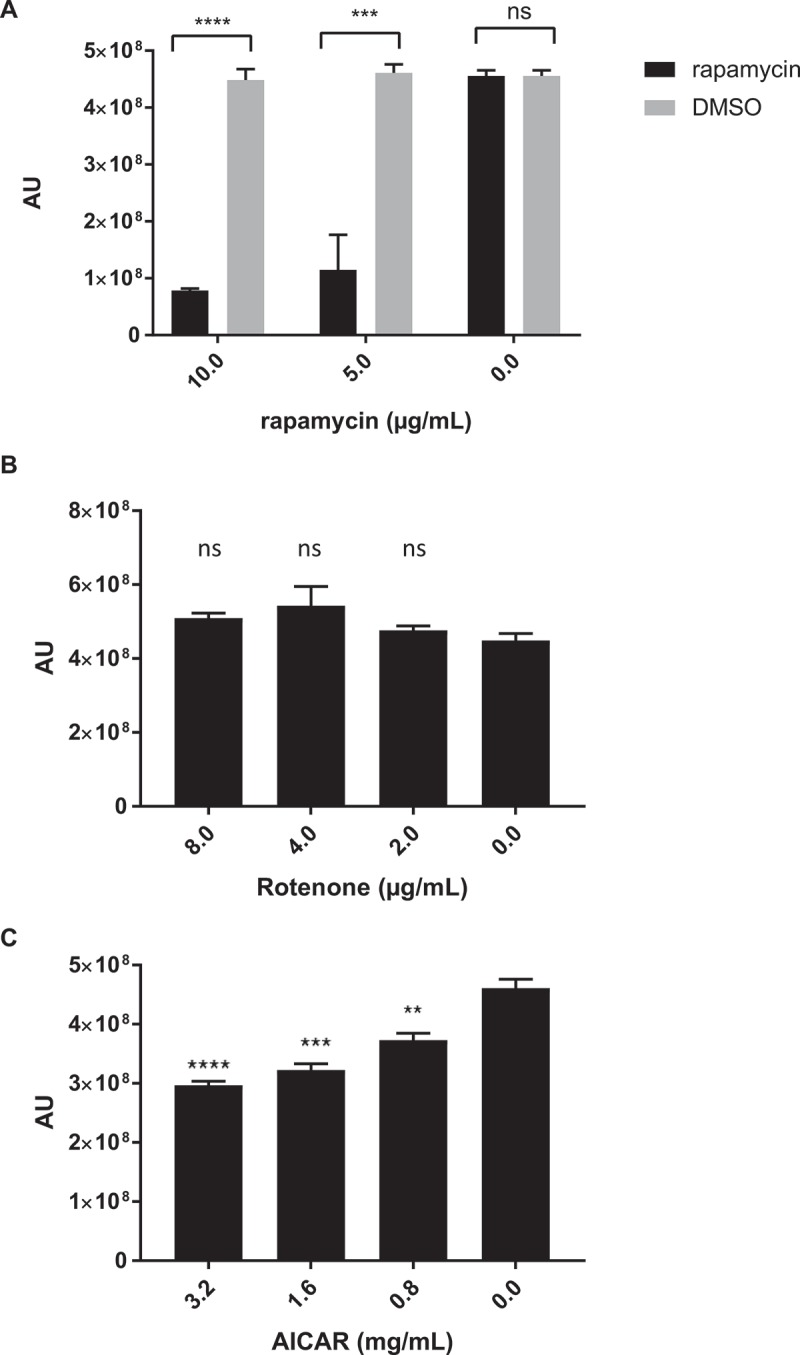
Inhibition of mTORC1 complex and AMPK, but not complex I, demonstrates anti-*C. glabrata* activity. Wild type *C. glabrata* were incubated with mTORC1 inhibitor rapamycin (A), mitochondrial complex I inhibitor rotenone (B), and AMPK agonist AICAR (C), in the presence of Prestoblue for 18h at 30°C. Yeast growth was expressed as arbitrary units for Prestoblue fluorescence (AU). *** denotes p ≤ 0.001, **** p ≤ 0.0001, ns = not significant. Data represent 3 independent experiments.

Previous studies have linked biguanide activity to mTOR, an essential regulator for growth and survival that is highly conserved among mammalian cell types. To assess if the antifungal effects of biguanides may also be mTOR related, chemical inhibition of mTORC1 with rapamycin was examined. Rapamycin exhibited a dose-dependent growth inhibition against *C. glabrata* with an MIC_50_ of 5–10 µg/mL (Figure 3(A)). Fractional inhibitory concentration index (ƩFIC) for rapamycin and metformin combinations was determined as a measurement of drug interaction between the two compounds. The combination is considered synergistic when the ΣFIC is ≤ 0.5, non-synergistic when the ΣFIC is ≥ 0.5 and ≤ 4, and antagonistic when ΣFIC is ≥ 4 [28,29]. Rapamycin and metformin ΣFIC was 0.84 ± 0.09, suggesting non-synergistic interaction between the two compounds. We then explored other upstream regulators of mTORC1, such as adenosine monophosphate-activated protein kinase (AMPK), an mTORC1 inhibitor, and alterations in cellular ATP levels. There was no effect on *C. glabrata* growth observed following treatment with the mitochondria complex I inhibitor rotenone (Figure 3(B)), but there was a small but statistically significant dose dependent decrease in *C. glabrata* growth following treatment with the AMPK agonist 5-Aminoimidazole-4-Carboxamide-1-Beta-D-Ribofuranoside (AICAR) (Figure 3(C)).

### Metformin enhances activity of multiple classes of antifungals

We further explored the ability of biguanides to augment the activity of FDA-approved, existing antifungal therapies used for the treatment of *C. glabrata* infection. We examined combinations of three biguanides: metformin, phenformin, and buformin, with four antifungals: voriconazole, fluconazole, amphotericin B deoxycholate, and micafungin, and we determined ƩFIC for all 12 possible combinations (Table 2). All three biguanides had ƩFICs in the range of 0.5 < ƩFIC < 2 with voriconazole, fluconazole, and amphotericin. Biguanide combinations with micafungin, however, was not as effective, with ƩFICs consistently > 2.

**Table 2.** ƩFIC of biguanides and antifungals.

	Voriconazole	Amphotericin B	Fluconazole	Micafungin
Metformin	0.61 ± 0.04	0.74 ± 0.03	0.63 ± 0.16	2.67 ± 0.33
Phenformin	0.94 ± 0.03	0.69 ± 0.11	1.02 ± 0.07	3 ± 0
Buformin	0.94 ± 0.12	0.80 ± 0.12	1.08 ± 0.08	2.33 ± 0.33

**Figure 4.**
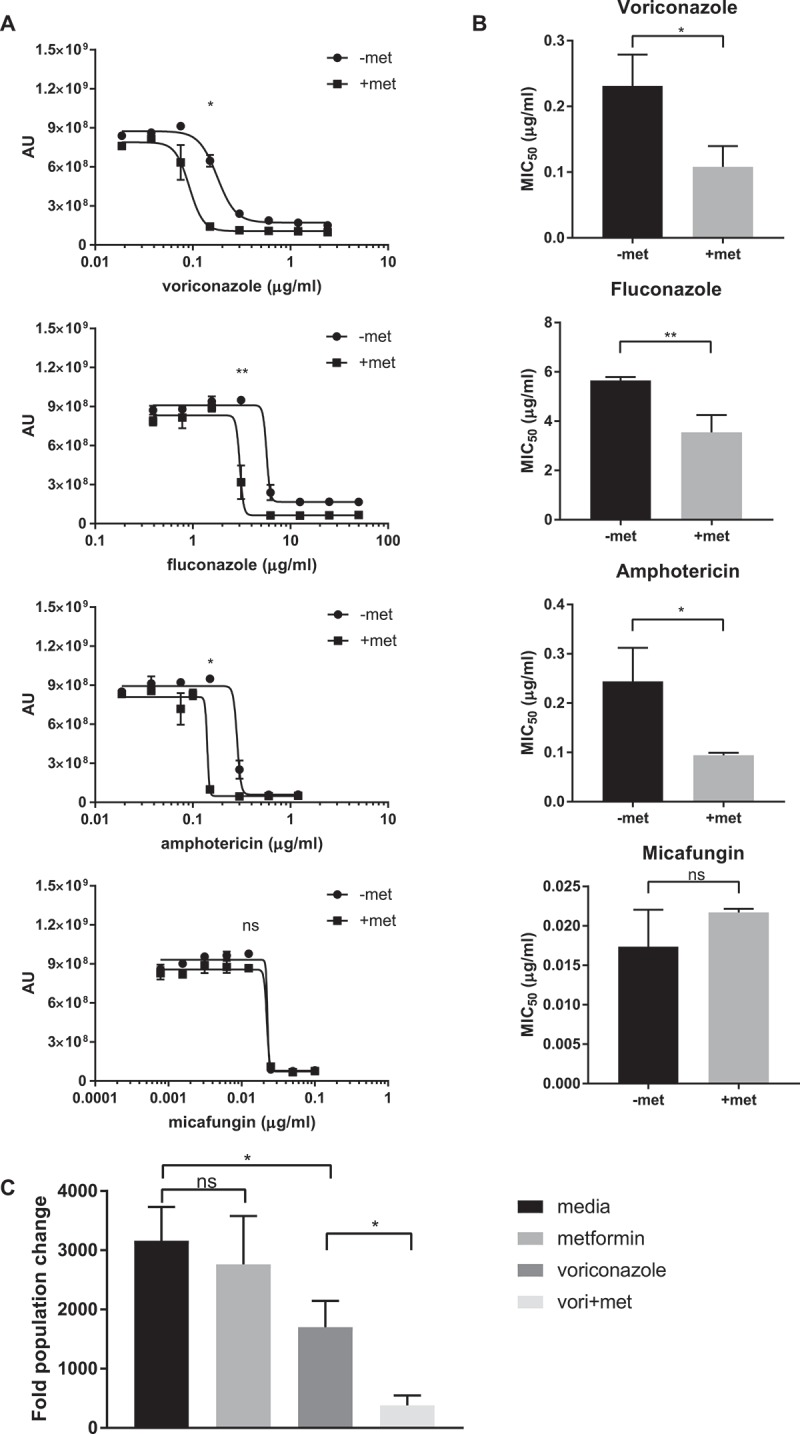
Combination therapy of metformin with common antifungal agents reduces *C. glabrata* activity. (A) Wild type *C. glabrata* treated with 6.5 mg/mL metformin in combination with voriconazole, fluconazole, amphotericin or micafungin in the presence of Prestoblue for 18hrs at 30°C. AU represent Prestoblue fluorescence unit. Data points were fitted to a four-parameter logistic curve. (B) MIC_50_ were determined from four-parameter logistic curves comparing control antifungal drug with metformin combination. (C) *C. glabrata* was treated with voriconazole, metformin, or the combination for 18hrs, resuspended and plated onto YPD agar plates for 24-48hrs at 30°C. Data are plotted as fold population change compared to initial inoculum. * denotes p ≤ 0.05, ** p ≤ 0.01. Data represent 3 independent experiments.

By introducing 6.5 mg/mL of metformin, a concentration without effect alone, to wild type *C. glabrata*, we were able to control yeast growth with lower concentrations of specific antifungal agents (Figure 4(A)). 6.5 mg/mL of metformin significantly lowered *C. glabrata* MIC_50_ to antifungals when combined with voriconazole, fluconazole, and amphotericin (Figure 4(B)). In contrast, combination of metformin with micafungin had no influence on the micafungin MIC_50_. The enhanced antifungal activity of metformin-voriconazole combination was confirmed by CFU assay (Figure 4(C)). Treatment with 6.5 mg/mL metformin and 40 ng/mL voriconazole reduced CFUs by 88% compared to *C. glabrata* treated with voriconazole alone.

### Metformin slows *C. glabrata* growth by prolonging yeast doubling time

**Figure 5.**
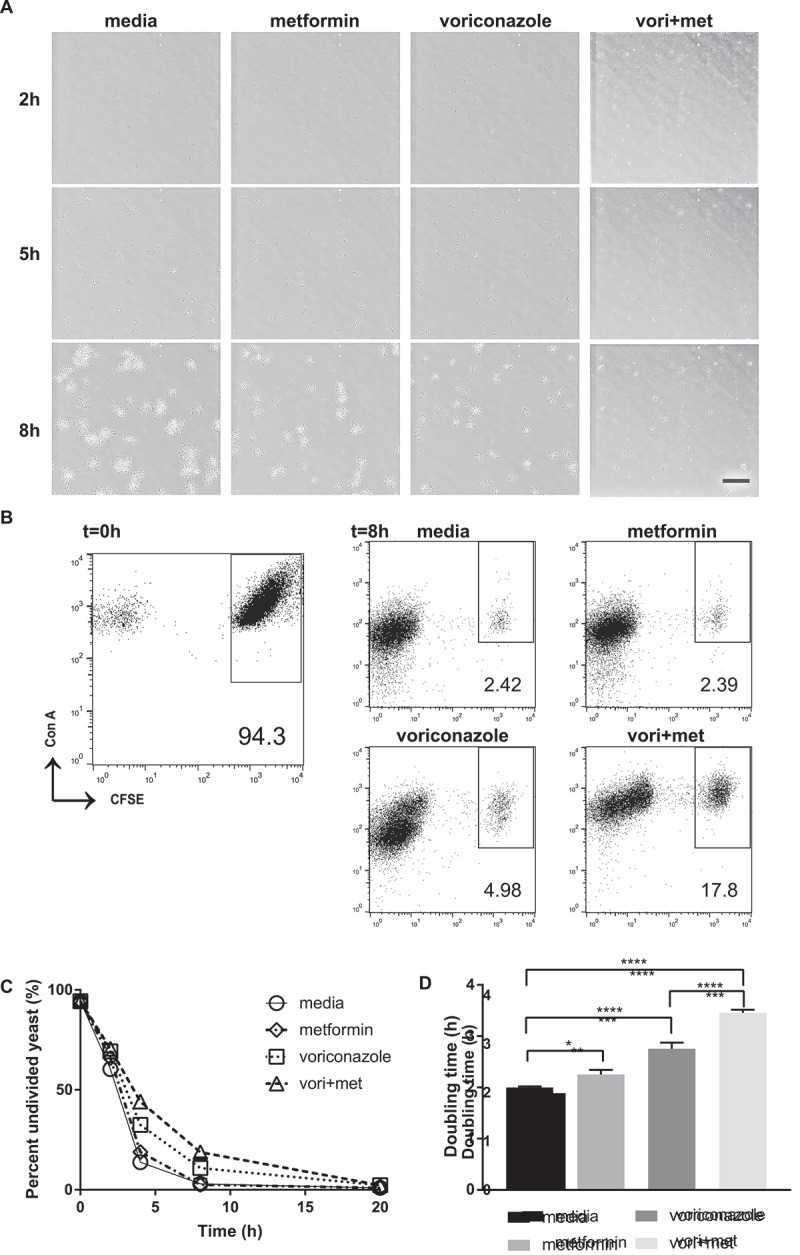
Metformin reduces *C. glabrata* proliferation by slowing division rate. (A) Time lapse microscopy of live wild type *C. glabrata* over 20hrs in a humidified microscopy chamber at 30°C. Images show frame at 8hrs (supplemental video displays entire time course). Scale bar represents 50µm. (B) Flow cytometry division measurement of CFSE-labeled *C. glabrata*. CFSE-bright undivided yeast population shown at t = 0 is then incubated in media, metformin (6.5mg/ml), voriconazole (40µg/mL) or combination. Percent (scatter plot inset) of undivided CFSE-bright population was determined over time indicated. Representative scatter flow plots shown at 8hrs, and (C) percent undivided population shown over time. (D) *C. glabrata* doubling time was determined from flow cytometry CFSE measurements for yeast incubated in media, metformin, voriconazole and combination treatment. * denotes p ≤ 0.05, **** p ≤ 0.0001. Data represent a minimum of 3 independent experiments.

To deepen our understanding of the process by which metformin affects *C. glabrata* growth overtime, we performed time-lapse microscopy with live *C. glabrata* dividing in the presence of metformin, voriconazole, or metformin-voriconazole combination treatment. While 6.5 mg/mL of metformin or 40 ng/mL voriconazole alone did not significantly change the rate of *C. glabrata* growth, combination treatment of metformin and voriconazole led to a drastic decrease in *C. glabrata* growth rate (Supplemental movie 1). Compared to *C. glabrata* that was untreated or treated with either metformin or voriconazole alone, *C. glabrata* grown in the combination of metformin-voriconazole exhibited slowed growth and smaller yeast clusters (Figure 5(A)). Our results suggest that the antifungal activity of metformin may be due to decreased rate of yeast division.

To further quantify these microscopic observations, we used a CFSE assay determine *C. glabrata* doubling time. Log phase *C. glabrata* were stained with CFSE as a fluorescent tracer of division. Yeast cells directly stained with CFSE are high in fluorescence intensity. During each cell division, percent of the undivided cell population decreases as additionally divided yeast cell numbers increase. Cells that have undergone division can be easily distinguished from undivided cells based upon CFSE fluorescence intensity. Using metformin-voriconazole combination treatment, we observed a higher percentage of bright undivided *C. glabrata* cells consistent with inhibition of yeast cell division as compared to no drug control (Figure 5(B,C)). The doubling time of *C. glabrata* increased significantly in the presence of metformin. Metformin alone significantly prolonged *C. glabrata* doubling time to 2.3h compared to untreated yeast at 2h, while metformin-voriconazole combination treatment prolonged doubling time to 3.5h (Figure 5(D)).

### Metformin decreases antifungal MIC_50_ in drug-resistant *C. glabrata* strains

**Figure 6.**
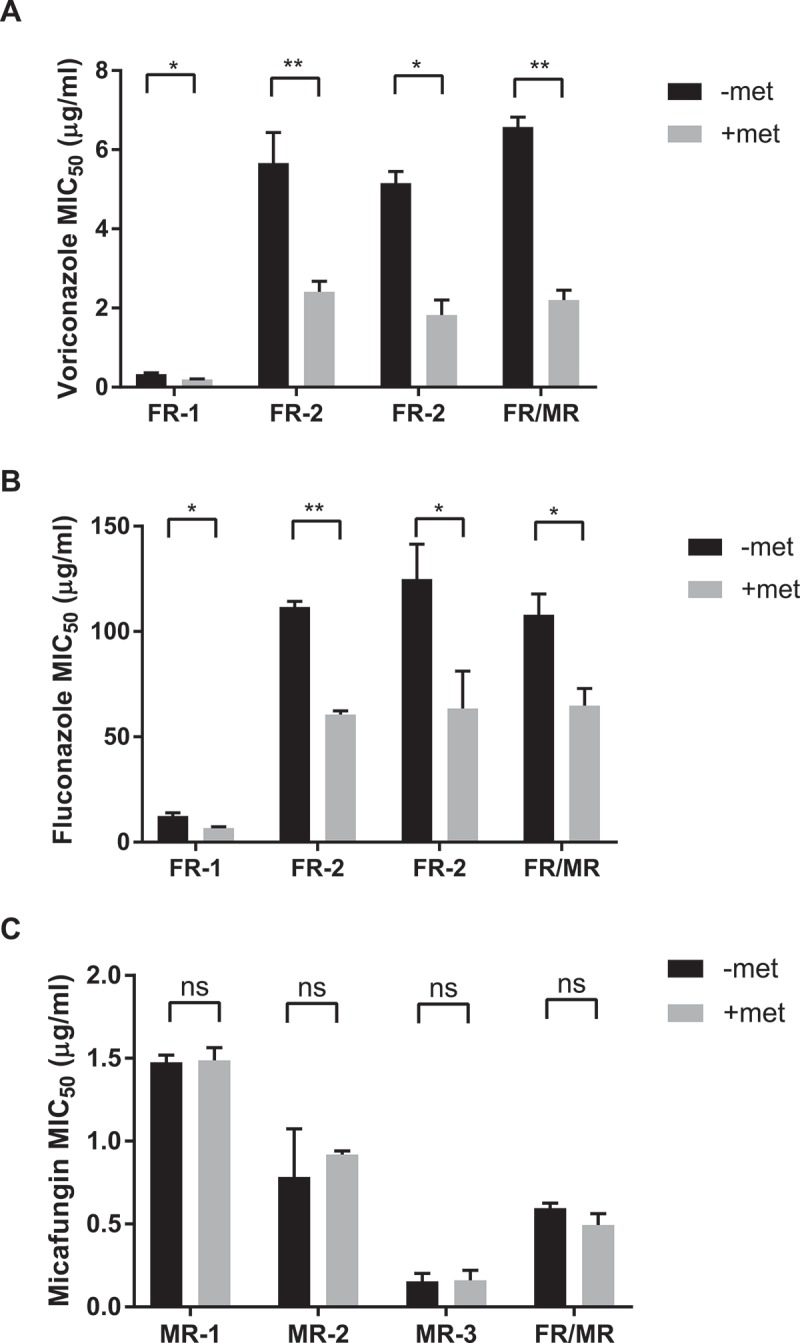
Metformin enhances antifungal activity against drug resistant *C. glabrata* isolates. Fluconazole-resistant clinical *C. glabrata* isolates were incubated with 3.9 mg/mL of metformin and voriconazole (A) or fluconazole (B). The MIC_50_ to each antifungal agent was determine with and without metformin. (C) Echinocandin and fluconazole double-resistant strain FR/MR was incubated with metformin and increasing amounts of micafungin. Micafungin MIC_50_ was determined for strain FR/MR with and without metformin. * denotes p ≤ 0.05, ** p ≤ 0.01. Data represent a minimum of 3 independent experiments.

Since combining metformin with antifungals enhanced drug efficacy, we sought to determine if metformin can increase the antifungal susceptibility of drug-resistant strains of *C. glabrata*. Seven strains of resistant *C. glabrata* clinical isolates were subjected to combinations of metformin and antifungal agents. Of the isolated strains used, 3 isolates (FR-1, FR-2, FR-3) are fluconazole-resistant with moderate voriconazole resistance, 3 isolates (MR-1, MR-2, MR-3) are micafungin-resistant, and a single isolate, FR/MR, is fluconazole and micafungin double resistant. We established that all seven isolates are susceptible to metformin, with metformin MIC_50_ around 6.5 mg/mL, similar to wild type control. Isolate susceptibility to voriconazole, fluconazole, and micafungin was tested in the presence and absence of metformin. With metformin treatment, all 4 azole-resistant clinical isolates demonstrated a lowered antifungal MIC_50_ to voriconazole and fluconazole (Figure 6(A,B)). For the micafungin-resistant isolates as well as the double resistant FR/MR isolate, addition of metformin had no influence on micafungin MIC_50_ (Figure 6(C)).

## Discussion

The metformin-induced growth inhibition pathway is conserved across a wide variety of organisms [27]. Despite the broad number of species studied, the effect of biguanides on fungal species has not previously been examined. In this study, we evaluated the antifungal properties of biguanides, both alone, and in combination with common antifungal agents. Our studies show that three biguanides – metformin, phenformin, and buformin – exhibit dose-dependent inhibition of *C. glabrata* as well as many other *Candida* species. In biguanide-antifungal combination therapies, the three biguanides combine favorably with voriconazole, fluconazole, and amphotericin, with ƩFICs ranging from 0.6 to 1. That these three biguanides demonstrate the same growth-inhibition and the same combination effect point to a likely common antifungal mechanism.

A significant limitation of these data is that the effective biguanide concentrations are beyond those normally achieved *in vivo*, in patient plasma [30,31]. The maximal approved total daily dose of metformin for treatment of diabetes is 35 mg/kg body weight, which results in a plasma concentration of 5–9 mg/L [32]. Our studies utilize concentrations significantly higher, although use of control drug in this concentration range did not show toxicity, suggesting that the antifungal response exerted by biguanides are class specific. Limited biguanide entry into *C. glabrata* as compared to mammalian cells is one possible factor leading to the need for high drug levels. Even among human cells, intracellular metformin concentrations differ widely, preferentially accumulating in liver tissues as a result of high expression of the organic cation transporter 1 (OCT1), the main plasma-membrane metformin transporter [33]. Overexpression of OCT1 can increase metformin uptake drastically [34]. On the other hand, studies investigating the role of metformin in cancer cells, that lack OCT1 expression, often require concentrations similar to those used in our study [23,33]. The lack of appropriate membrane transporters in *C. glabrata* may hinder metformin uptake, thereby requiring the extreme concentrations used in these studies. These observations highlight the need for structural modifications of biguanides that may allow for heightened antifungal activity.

Our studies using time-lapse microscopy and flow cytometric yeast division analysis demonstrate a continuous slowing of cell division in metformin-treated *C. glabrata*. This reduction in growth rate points towards modulation of cellular metabolism or cell cycle activity as possible targets of the antifungal activity of metformin. Multiple possible molecular mechanisms of actions may contribute to biguanide antifungal activity. Of particular interest are mTORC1 pathways. Biguanides can limit mitochondrial complex I activity, lowering intracellular ATP and in turn elevating AMP [35]. AMP accumulation prompts (AMPK) to deactivate mTORC1, while ATP deprivation causes changes in the nuclear pore complex conformation, restricting localization of mTORC1-activating Rag proteins [27,36]. Both pathways result in inhibition of mTORC1, which is a highly conserved signaling hub shared between fungi and mammalian cells [37]. The mTORC1 complex is involved in regulation of a vast range of cellular protein target, having downstream effects in growth, proliferation, oxidative stress, and lipid metabolism [38–40]. The pathway has also been previously linked to antifungal activity with anti-*Mucorales* activity [41]. We demonstrate mTOR inhibition also leads to reduction in *C. glabrata* metabolic activity, and that rapamycin-metformin combinations show non-synergistic drug interactions. Similarly, in nematode growth studies, such as *C. elegans*, biguanide-dependent inhibition of mTORC1 resulted in limited growth [27]. In contrast, the mitochondria complex I inhibitor, rotenone, was effective in limiting *C. elegans* body size, but exerts no effect in *C. glabrata*. The AMPK agonist AICAR had no effect on *C. elegans*, but demonstrated a small, but statistically significant reduction in metabolic activity in *C. glabrata*, suggesting AMPK-dependent mTOR inhibition contributes to growth reduction in yeast. Additionally, the non-synergistic drug interactions between rapamycin and metformin suggest alternate metabolic pathways to mTOR may also be involved in biguanide activity. Both observations require further studies to delineate the precise biguanide-dependent molecular mechanisms in *Candida*.

Our data also suggest augmentation of current antifungals by metformin. Given that azole drugs function by inhibition of lanosterol 14α-demethylase in the ergosterol biosynthesis pathway [42,43] and polyenes by binding to ergosterol, these data raise sterol synthesis as a possible mechanism of metformin-induced antifungal activity. Interestingly, the lack of biguanide activity with echinocandins, potent inhibitors of beta-glucan synthase [9], may either stem from the involvement of two distinct and independent pathways, or from influencing the same precise biochemical process. Biguanides can also alter ATP concentration, in turn, affecting ATP-binding cassette (ABC) transporter activity [44]. ABC transporters are a major etiology of azole resistance in *C. glabrata* isolates [44] and disruption of azole efflux could allow for more concentrated intracellular azoles. Micafungin, on the other hand, is not an efflux pump substrate [45], potentially offering another explanation for the lack of effect with biguanide-micafungin combinations.

In summary, our findings suggest that *in vitro*, metformin, as well as other biguanides, demonstrate antifungal activity against *C. glabrata*. Biguanides possess antifungal alone but can also enhance antifungal efficacy of azole and polyene agents. Importantly, from a therapeutic perspective, biguanides lower the resistance of antifungal-resistant isolates to fungal drugs, specifically polyene and azole resistant isolates, suggesting combinatorial therapy may assist with clinical drug resistance. We have yet to explore the antifungal properties of biguanides *in vivo* as the high biguanide concentrations are currently unachievable. Intriguingly, in models of *Mycobacteria tuberculosis* infection, metformin enhanced host leukocyte activity, including metformin-associated reactive oxygen species production and autophagy promotion enhancing pathogen elimination [46,47]. Whether metformin could enhance the antifungal activity of human leukocytes is not known, however it is possible that in addition to the direct antifungal effects of metformin, there may be an effect on antifungal immunity. While the molecular mechanisms of biguanide antifungal activity require closer examination, these data suggest that biguanides offer a potential new therapeutic modality alone or in combination antifungal therapy.

## Materials and methods

### Reagents

Metformin hydrochloride was purchased from Cayman Chemicals. Fluconazole, ampicillin sodium, rapamycin, and other biguanide hydrochlorides including phenformin and buformin were purchased from Sigma. Clinical grade formulations of the antifungal agents voriconazole, amphotericin B deoxycholate, and micafungin were purchased from the Massachusetts General Hospital (MGH) clinical pharmacy. All reagents were dissolved in distilled water or DMSO according to the manufacturer’s recommendation, and stored at −20°C. Cell culture media include liquid YPD (1% yeast extract, 2% peptone, 2% dextrose); YPD agar (1% yeast extract, 1% peptone, 2% dextrose, 2% agar); RPMI-MOPS (RPMI 1640 containing 2% glucose and 0.165M MOPS, buffered at pH7); and complete RPMI (RPMI 1640 w/2mM L-glutamine, 10% heat-inactivated fetal bovine serum, 1% penicillin-streptomycin).

### Candida strains and culture conditions

Wild type *C. glabrata* and wild type SC5314 *C. albicans* were purchased from the American Type Culture Collection (ATCC, Manassas, VA). Clinical strains of other *Candida* species were obtained from the MGH microbiology lab or Dr. Dimitrios Kontonyanis (MDAnderson Cancer Center). Drug resistant isolates included one dose-dependent fluconazole resistant strain (Strain FR-1), two fluconazole-resistant strains (FR-2, FR-3), three micafungin-resistant strains (MR-1, MR-2, MR-3), and one fluconazole-micafungin double-resistant strain (FR/MR), as determined by Clinical Laboratory and Standards Institute (CSLI) criteria [48]. Antifungal susceptibility testing was confirmed by the MGH microbiology lab using microplate dilution and summarized in Supplemental Table 1. Yeast were grown overnight in liquid YPD at 30°C with shaking, washed three times in PBS after collection, counted with a Luna automated cell counter (Logos Biosystems), and resuspended in PBS at the desired inoculum.

### Drug susceptibility testing

Biguanide drug testing was adopted from CLSI microdilution antifungal testing [48]. *Candida* strains were incubated in RPMI-MOPS media at an inoculum of 200 yeasts per 100µl of media, and the indicated concentration of test drug. Prestoblue Cell Viability Reagent (Thermo Fisher) was added to 10% of the final volume. Cells were incubated at 35°C. After 20-24h of incubation, the MIC was determined based on fluorescence reading at 560/590nm using a SpectraMax i3x reader (Molecular Devices) [49]. The MIC_50_ was determined using GraphPad Prism 7 (GraphPad Software) non-linear curve fit equation. Log-transformed concentration values and Prestoblue dye fluorescence data were fitted to a four-parameter logistic equation, from which the concentration at 50% maximal value was extracted as the MIC_50_. To determine yeast viability after drug exposure and confirm Prestoblue measurements, colony forming unit (CFU) assays were performed. After incubation as above, yeasts were serially diluted in distilled water and plated on YPD plates. CFU were determined manually after 48 hours of incubation at 30°C. Initial inoculum was also plated to confirm the starting number of yeast cells.

### Fractional Inhibitory Concentration (FIC) index determination

Mode of drug interaction in combinations of biguanides and antifungals were determined by assessing the fractional inhibitory concentration index (ΣFIC) through the checkerboard method [28]. Following inoculation, checkerboard plates were incubated at 35°C and read at 560/590nm fluorescence after 18 hours. MIC was defined as the lowest concentration of drug or drug combination that completely inhibited growth of the organism as determined by fluorescence reading. FIC and FIC indexes (ΣFIC) were determined as described previously, where the combination is considered synergistic when the ΣFIC is ≤ 0.5, non-synergistic when the ΣFIC is ≥ 0.5 and ≤ 4, and antagonistic when ΣFIC is ≥ 4 [28,29].

### Flow cytometric analysis of yeast doubling time

Wild type *C. glabrata* proliferation was determined by carboxyfluorescein diacetate succinimidyl ester (CFSE) labeling. Similar to cell wall labeling with FITC [50], CFSE allows for tracing of multiple generations of yeast division by dye dilution, giving rise to highly fluorescent undivided cells and divided cell populations with lower fluorescence intensity. Wild type *C. glabrata* were grown to log phase in liquid YPD, washed in PBS, and stained with 25µg/mL CFSE (Sigma) for 30 min at 30°C on a vertical rotator. Yeasts were washed in PBS containing 2% bovine serum albumin (BSA) to remove excess CFSE. Stained yeast cells were passed through a 25G 7/8-inch needle to dissociate clumped cells.

CFSE stained *C. glabrata* was inoculated in MOPS-RPMI media with desired concentrations of biguanide and antifungal agents. At the desired time points, yeast cells were washed and fixed in 3.7% formalin and labeled with 3µg/mL concanavalin A conjugated to Alexa Fluor 647 (Thermo Fisher). Flow acquisition was done with a FACS Calibur flow cytometer (Becton-Dickinson), using CellQuest software (Becton-Dickinson). After expressing ConA-647 on the y-axis and CFSE on the x-axis, the percentage of CFSE bright undivided population was determined using FlowJo 10 software (FlowJo, Ashland, OR). To determine yeast doubling time, percentage of undivided population over time was fitted to a non-linear one phase exponential decay curve fit equation in GraphPad Prism 7.

### Microscopy

Wild type *C. glabrata* was plated onto 8-chambered coverslip slides (LabTek, Thermo Scientific) and allowed to proliferate in MOPS-RPMI in the presence of metformin, voriconazole, or metformin-voriconazole combined treatment. Slides were mounted on a Nikon Ti-E inverted microscope equipped with an EM-CCD camera (Hamamatsu, C9100-13). For live cell imaging, the microscopy chamber, housed in an environmental chamber, was humidified and set to 30°C [51]. Images were acquired using MetaMorph software (Molecular Devices).

### Statistics

Statistical calculations were performed using GraphPad Prism 7 software. Data were analyzed by two-tailed unpaired t test, or one-way ANOVA test, where appropriate, and were considered significant when p ≤ 0.05.
