# Supplementary material for: Biguanides enhance antifungal activity against Candida glabrata
Source: Virulence. 2018 Aug 1;9(1):1150–62. doi: 10.1080/21505594.2018.1475798 (PMC6086317; doi:10.1080/21505594.2018.1475798)
Supplement: Supplemental Material [file kvir-09-01-1475798-s001.docx]

**Supplemental Materials**

**Supplemental table 1.** *C. glabrata* isolates and antifungal drug MIC (µg/ml).

|  | Voriconazole | Fluconazole | Amphotericin B | Micafungin |
| --- | --- | --- | --- | --- |
| FR-1 | 0.5 | 16 | 1 | 0.015 |
| FR-2 | 0.5 | 64 | 1 | 0.015 |
| FR-3 | 1 | 64 | 1 | 0.03 |
| MR-1 | - | 8 | 1 | 2 |
| MR-2 | - | 8 | 0.5 | 1 |
| MR-3 | 0.5 | 32 | 1 | 0.25 |
| FR/MR | 2 | 64 | 1 | 1 |
| Wild type | 0.06 | 2 | 0.5 | 0.015 |

**Supplemental video 1.** Time lapse microscopy of live wild type *C. glabrata* treated with metformin, voriconazole, or the combination, over 20hrs in a humidified microscopy chamber at 30°C.
